# Evaluation of Diabetes Care Performance in Cambodia Through the Cascade-of-Care Framework: Cross-Sectional Study

**DOI:** 10.2196/41902

**Published:** 2023-06-22

**Authors:** Vannarath Te, Srean Chhim, Veerle Buffel, Wim Van Damme, Josefien van Olmen, Por Ir, Edwin Wouters

**Affiliations:** 1 School of Public Health National Institute of Public Health Phnom Penh Cambodia; 2 Health Policy Unit, Department of Public Health Institute of Tropical Medicine (Antwerp) Antwerp Belgium; 3 Department of Family Medicine and Population Health University of Antwerp Antwerp Belgium; 4 Technical Office National Institute of Public Health Phnom Penh Cambodia; 5 Centre for Population, Family & Health, Department of Sociology University of Antwerp Antwerp Belgium; 6 Centre for Health Systems Research & Development University of the Free State Bloemfontein South Africa

**Keywords:** diabetes, cascade of care, implementation research, population-based survey, care continuum, mobile phone

## Abstract

**Background:**

Cambodia has seen an increase in the prevalence of type 2 diabetes (T2D) over the last 10 years. Three main care initiatives for T2D are being scaled up in the public health care system across the country: *hospital-based care*, *health center–based care*, and *community-based care*. To date, no empirical study has systematically assessed the performance of these care initiatives across the T2D care continuum in Cambodia.

**Objective:**

This study aimed to assess the performance of the 3 care initiatives—individually or in coexistence—and determine the factors associated with the failure to diagnose T2D in Cambodia.

**Methods:**

We used a cascade-of-care framework to assess the T2D care continuum. The cascades were generated using primary data from a cross-sectional population-based survey conducted in 2020 with 5072 individuals aged ≥40 years. The survey was conducted in 5 operational districts (ODs) selected based on the availability of the care initiatives. Multiple logistic regression analysis was used to identify the factors associated with the failure to diagnose T2D. The significance level of *P*<.05 was used as a cutoff point.

**Results:**

Of the 5072 individuals, 560 (11.04%) met the definition of a T2D diagnosis (fasting blood glucose level ≥126 mg/dL and glycated hemoglobin level ≥6.5%). Using the 560 individuals as the fixed denominator, the cascade displayed substantial drops at the testing and control stages. Only 63% (353/560) of the participants had ever tested their blood glucose level in the last 3 years, and only 10.7% (60/560) achieved blood glucose level control with the cutoff point of glycated hemoglobin level <8%. The OD hosting the coexistence of care displayed the worst cascade across all bars, whereas the OD with hospital-based care had the best cascade among the 5 ODs. Being aged 40 to 49 years, male, and in the poorest category of the wealth quintile were factors associated with the undiagnosed status.

**Conclusions:**

The unmet needs for T2D care in Cambodia were large, particularly in the testing and control stages, indicating the need to substantially improve early detection and management of T2D in the country. Rapid scale-up of T2D care components at public health facilities to increase the chances of the population with T2D of being tested, diagnosed, retained in care, and treated, as well as of achieving blood glucose level control, is vital in the health system. Specific population groups susceptible to being undiagnosed should be especially targeted for screening through active community outreach activities. Future research should incorporate digital health interventions to evaluate the effectiveness of the T2D care initiatives longitudinally with more diverse population groups from various settings based on routine data vital for integrated care.

**Trial Registration:**

International Standard Randomized Controlled Trials Number (ISRCTN) ISRCTN41932064; https://www.isrctn.com/ISRCTN41932064

**International Registered Report Identifier (IRRID):**

RR2-10.2196/36747

## Introduction

### Background

In 2021, globally, 1 in 10 adults aged 20 to 79 years was living with type 2 diabetes (T2D) [1]. Similar to other countries in the Western Pacific region—the World Health Organization (WHO) region with the highest number of adults living with T2D (206 million) [1]—Cambodia has been severely affected by the T2D epidemic. In 2016, the prevalence rate of T2D in this lower–middle-income country was 9.6% among adults aged 18 to 69 years, signifying a significant increase compared with the prevalence rate in 2010 (2.9% among adults aged 25-64 years) [2]. As a response to the increased burden of chronic conditions, including T2D, across the globe, the WHO adopted the innovative care for chronic conditions (ICCC) framework as a road map for countries, regardless of income level, to transform their health systems toward better care for chronic conditions [3].

The incurable nature of T2D, along with its chronicity and silent progression, requires the condition to be diagnosed as early as possible and managed properly and promptly on a regular basis by patients, caregivers, and health care professionals to prevent or delay complications [3]. Care for T2D relies not only on medical interventions provided by health care professionals but also on high-quality and continuous self-management [4]. A systematic review shows that the quality of T2D care in low- and middle-income countries (LMICs) in Asia and the Middle East has been reported to be limited, with the care goals recommended in the evidence-based guidelines not being met [5]. The WHO promotes the adoption of integrated care for disease management in the health system as outlined in the ICCC framework [3], which is evidence based in improving blood glucose level control [6,7], to fill the gaps.

Likewise, care for T2D in Cambodia has been limited among the population at risk and people living with T2D. Many adults (more than two-thirds of the population) have never had their blood glucose level tested, and more than half of those living with T2D are not receiving treatment [2].

Cambodia’s health system is pluralistic—both public providers and private providers (including nonprofit organizations) provide care for T2D in the country. The ministry of health is in charge of the public health providers, which are organized on a district health system model and guided by the primary health care approach [8]. In this model, an operational district (OD) usually comprises a referral hospital providing secondary care and 10 to 25 health centers providing primary care with support from community health workers. The public providers mainly cover health prevention activities by providing primary health care for people with infectious diseases (such as HIV infection and AIDS, tuberculosis, and malaria) and focusing on maternal and child health, leaving care for chronic conditions, including T2D, to be provided mainly by private providers [9]. A self-reported survey on availability of T2D services at primary care facilities indicated that only approximately 1 in 5 health centers reported providing T2D services [10].

To improve the availability of integrated care for T2D, three main care initiatives for T2D are currently being scaled up across the 103 ODs in Cambodia [11]: (1) *hospital-based care*, (2) *health center–based care*, and (3) *community-based care*. In 2019, the ministry of health approved a national standard operating procedure for the management of T2D and hypertension in primary care in an attempt to integrate these 3 care initiatives for the T2D and hypertension care continuum in which health centers provide continuity and coordination across the care levels in the OD [11]. This standard operating procedure was adapted from the WHO package of essential noncommunicable disease interventions (WHO PEN) [12]—we refer to health centers implementing the standard operating procedure as the WHO PEN health centers.

Hospital-based care is standard care at the referral hospitals that provide ambulatory care for serious or complicated T2D cases. In 2018, with support from the ministry of health, 29 of the 117 referral hospitals provided exclusive care for T2D and hypertension at separate noncommunicable disease (NCD) clinics [13]. Health center–based care is implemented at the WHO PEN health centers. They are allowed to take care of mild or stable T2D cases without complications, with the diagnosis confirmed and treatment initiated at the referral hospital. In early 2020, only 86 of the 1221 health centers had implemented the WHO PEN program [13]. Community-based care is implemented through peer educator networks run by a Cambodian nongovernmental organization called MoPoTsyo. The networks offer (1) self-management support to patients through peer educators who have been diagnosed with T2D themselves, (2) laboratory tests, (3) physician consultations, and (4) low-cost medicines through a revolving drug fund program [14]. Each peer educator is responsible for a health center’s catchment area, with populations ranging from 10,000 to 20,000 [8]. By 2019, MoPoTsyo had 255 peer educators trained to serve >40,000 patients [14]. Detailed descriptions of the 3 care initiatives have been provided in a study protocol [15].

The coexistence of the 3 care initiatives—combining hospital-, health center–, and community-based care components—in an OD could potentially produce the ideal context for integrated care as described in the ICCC framework [3]. However, for the care initiatives to be integrated and thus for these 3 care initiatives to strengthen each other, necessary information has to be shared, and resources have to be coordinated in an effective and efficient manner [16].

To our knowledge, no empirical study has assessed the performance of the aforementioned 3 care initiatives—either individually or in coexistence—across the T2D care continuum in Cambodia. We used the test-treat-retain cascade of care as adapted from the HIV program to assess the T2D care continuum [17]. This method allowed us to document how many patients were lost along the care continuum with regard to testing, diagnosis, retention in care, receiving treatment, and achieving good control of their health condition. In other LMICs, limited existing studies have pooled secondary data from cross-sectional surveys to generate countrywide cascades of care [18-20] as an approach to assess health system performance to meet the T2D care continuum goals. A systematic assessment of the performance of the different care initiatives—either individually or in coexistence—currently being scaled up in Cambodia is not yet available.

### Objectives

This study aimed to address the research gap by assessing the performance of the aforementioned care initiatives either individually or in coexistence with the cascade-of-care framework using primary data from a population-based survey and determining the factors associated with the undiagnosed status of T2D among the population.

## Methods

### Study Design

The study was part of a population-based survey conducted in 2020. It was a cross-sectional study involving 5072 individuals aged ≥40 years [15]. A detailed explanation of the study design was included in the study protocol [15].

### Study Setting

Five ODs were purposively selected to assess the performance of the aforementioned care initiatives—individually or in coexistence. OD Samrong in Oddar Meanchey province provided hospital-based care at the NCD clinic of the referral hospital—the only public provider for T2D care in the OD at the time of the study. People with T2D visited the physician for a medical consultation (prescriptions and medicines were provided) on a monthly appointment basis. The second selected OD was OD Pearaing in Prey Veng province. This OD began implementing health center–based care in 2015. Assigned staff at the WHO PEN health centers receive training to perform screening, provide follow-up care for mild and stable T2D cases, and offer health education and counseling on healthy behavior as part of cardiovascular disease risk factors management [11]. By structural design, the NCD clinic at the referral hospital is required to support the WHO PEN health centers. In this OD, at the time of the study, 8 of the 9 health centers were WHO PEN health centers (high coverage). The third selected OD was OD Sotr Nikum in Siem Reap province. This OD has been historically and substantially influenced by financial aid from various development partners and nongovernmental organizations. At the time of the study, 5 of the 25 health centers in the OD were WHO PEN health centers (low coverage), supported by a chronic disease clinic that provided treatment and care to both people with T2D and hypertension and those with HIV infection [21]—the clinic was essentially the NCD clinic of the referral hospital. Therefore, we consider this OD the host of health center–based care (with context). The fourth selected OD was OD Kong Pisey in Kampong Speu province. In this OD, the peer educator network provided community-based care. At the time of the study, none of the public providers in this OD formally offered care for people with T2D. MoPoTsyo made arrangements with the referral hospital to provide physician consultations for people with T2D in the network once a week. The fifth selected OD was OD Daunkeo in Takeo province—the only OD found to host all 3 care initiatives together across the 103 ODs in Cambodia. At the time of the study, hospital-based care in this OD was provided at the NCD clinic of the referral hospital, whereas 8 of the 15 WHO PEN health centers in this OD provided health center–based care. The peer educator network provided community-based care, but the network had already been handed over to the OD health authorities for governance. Table 1 summarizes the study settings.

**Table 1:** Selected provinces and operational districts (ODs) with different types of care initiatives.

Name of OD	Province	Existing care provision	Care initiative
Samrong	Oddar Meanchey	NCD^a^ clinic at referral hospital	Hospital-based care
Pearaing	Prey Veng	NCD clinic+WHO PEN^b^ (high coverage)	Health center–based care
Sotr Nikum	Siem Reap	NCD clinic+WHO PEN (low coverage)	Health center–based care with context
Kong Pisey	Kampong Speu	Peer educator network	Community-based care
Daunkeo	Takeo	NCD clinic+WHO PEN+peer educator network	Coexistence of care

^a^NCD: noncommunicable disease.

^b^WHO PEN: World Health Organization package of essential noncommunicable disease interventions.

### Study Participants and Recruitment

The target study participants were adults aged ≥40 years. This age group was targeted for T2D screening according to the national standard operating procedure [11]. The recruitment was processed via a 3-level procedure. First, within each OD, a list of villages affected by the care initiative was drawn up, and 44 villages were randomly selected. Second, 24 eligible households (ie, those containing at least 1 household member aged ≥40 years) were randomly selected from a list of all eligible households in the selected villages. Within the selected households, potential participants had to be (1) usual members of the household either staying in the house the night before the interview or not being absent for >6 months, (2) physically and mentally capable of answering questions, and (3) well-informed regarding the consent procedure for participation in the study. In the third step, 1 household member meeting the aforementioned eligibility criteria from each randomly selected household was randomly recruited into the study. Each selected participant was interviewed based on a preset questionnaire and their anthropometric measurements (blood pressure, body weight, height, and waist and hip circumferences) and biochemical measurements (fasting blood glucose [FBG] level, glycated hemoglobin [HbA_1c_] level, and creatinine level) taken. Data were digitally collected using the KoboToolbox system developed by the Harvard Humanitarian Initiative [22].

### Measures and Analytical Strategy

#### Primary Outcome of Interest

The main outcome of interest in this study was the cascade of care consisting of six bars: (1) the *prevalence* bar, (2) the *ever tested or screened* bar, (3) the *ever diagnosed* bar, (4) the *in care* bar, (5) the *in treatment* bar, and (6) the *under control* bar. A fixed denominator approach was used for constructing the cascades of care to identify the leakages between the stages of the care continuum [23]. Table 2 shows the definitions of each bar and describes the sources of the data extracted for the analysis.

**Table 2:** Definitions of the cascade bars for type 2 diabetes (T2D).

Bars of the cascade of care for T2D and definitions		Source of data extracted for analysis
**Prevalence of the target population living with T2D**		
	Participants having biochemical measurement of FBG^a^ (capillary plasma value) level ≥126 mg/dL (7 mmol/L) and HbA_1c_^b^ level ≥6.5% [18,24,25]	Measurement of FBG level Measurement of HbA_1c_ level
	Participants reporting the use of drugs for T2D, irrespective of their biomarker values	Response to the question, *Have you ever been told by a physician or other health worker that you have T2D?*
**Number of people in the target population with T2D ever tested for T2D**		
	Patients classified as living with T2D having had FBG level tested in the last 3 years	Response to the question, *Have you ever had your blood glucose level tested in the last 3 years?*
**Number of those tested ever diagnosed for T2D**		
	Tested patients with T2D reporting ever being told by a physician or other health worker that they have T2D	Response to the question, *Have you ever been told by a physician or other health worker that you have T2D?*
**Number of those diagnosed in care**		
	Patients diagnosed with T2D reporting receiving treatment or care for their conditions at least once in the past 12 months	Response to the question, *Did you get treatment or care for your T2D condition in the past 12 months?*
**Number of those in care receiving treatment**		
	Patients with T2D in care reporting using drugs for T2D or insulin in the past 2 weeks	Response to the question, *Are you currently receiving any of the following treatments for your T2D condition prescribed by a physician or other health care worker?* *Insulin* *Drugs (medication) that you have taken in the past 2 weeks*
	Patients with T2D in care reporting following advice to lose weight, stop smoking, perform physical exercise, and be on a special prescribed diet	Response to the question, *Are you currently receiving all of the following advice for your T2D condition prescribed by a physician or other health care worker?* *Special prescribed diet* *Advice to lose weight* *Advice to stop smoking* *Advice to start or perform more physical exercise*
**Number of those receiving treatment with T2D under control**		
	Patients with T2D in treatment having HbA_1c_ level <8% [18]	Measurement of HbA_1c_ level for known T2D

^a^FBG: fasting blood glucose.

^b^HbA_1c_: glycated hemoglobin.

#### Secondary Outcomes of Interest

The secondary outcomes of interest were the factors associated with the undiagnosed status of participants living with T2D. We defined *person with undiagnosed status* as a person having biochemical measurements of FBG level ≥126 mg/dL and HbA_1c_ level ≥6.5% in our study but never being told by a physician or other health worker that they had T2D.

#### Explanatory Variables

The explanatory variables for this analysis included demographic characteristics, socioeconomic status, and the care initiatives (either individually or in coexistence). The demographic characteristics consisted of (1) age in years (40-49, 50-59, or ≥60); (2) sex (male or female); (3) educational attainment (none, primary school, secondary school, or higher); and (4) socioeconomic status (poorest, poor, medium, rich, or richest), which was measured using a household wealth index. To obtain the household wealth index, each household was interviewed using a 20-item questionnaire adapted from the 2014 Cambodia Demographic Health Survey [26]. This tool has been validated and widely used to classify household socioeconomic class [27]. Finally, the care initiative settings included (1) hospital-based care, (2) health center-based care, (3) health center-based care with context, (4) community-based care, and (5) the coexistence of the 3 care initiatives.

#### Statistical Analysis

We produced bar charts of the T2D cascades of care in accordance with the definitions provided in Table 2. We used bivariate analyses to compare the proportion of participants living with T2D without a diagnosis by participant characteristics. Subsequently, a multiple logistic regression analysis was used to identify the factors associated with the undiagnosed status. As we had only a limited number of explanatory variables in the bivariate analysis, we included all these variables in our initial multiple logistic regression analysis, regardless of the significance level. We additionally used a backward elimination method. Variables with the highest *P* value were eliminated from the model one by one. We retained all variables with a significance level of *P*<.05 in the final model. The statistical software Stata (version 14.2; StataCorp LLC) was used to perform the statistical analyses [28].

### Ethics Approval

This study was approved by the National Ethics Committee for Health Research in Cambodia (NECHR; 105 NECHR) and by the institutional review board of the Institute of Tropical Medicine (Antwerp, Belgium; 1323/19). The study has also been registered as part of the Scale-up Integrated Care for Diabetes and Hypertension (SCUBY) project protocol at the ISRCTN Registry (ISRCTN41932064).

## Results

### Cascade of Care

**Figure 1:**
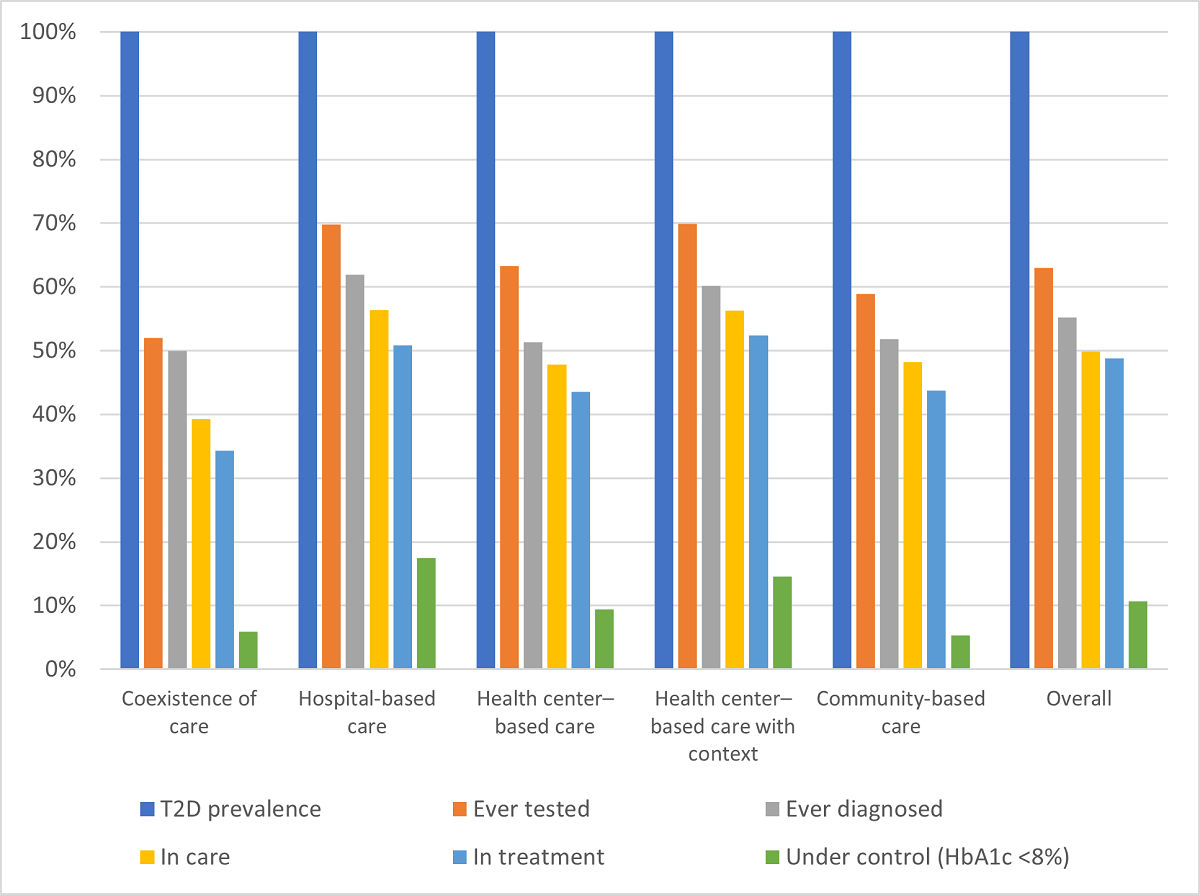
Type 2 diabetes (T2D) cascades of care by setting in 2020 in Cambodia. HbA_1c_: glycated hemoglobin.

Of the 5072 individuals participating in this study, 614 (12.11%) had raised blood glucose level (FBG level ≥126 mg/dL), and 560 (11.04%) met the definition of having T2D (Multimedia Appendix 1). Using the 560 individuals as the fixed denominator, we observed that 2 bars—*ever tested* and *under control*—had a substantial drop. Of the 560 individuals with T2D, only 353 (63%) had ever undergone a blood glucose level test in the last 3 years, 309 (55.2%) had ever been diagnosed as having T2D, 279 (49.8%) had received care in the past 12 months, and 273 (48.8%) had received insulin or antidiabetic medication in the past 2 weeks. In addition, only 130 (47.6%) of the 273 treated participants also received advice regarding a prescribed diet, weight loss, smoking cessation, and physical exercise. Only 10.7% (60/560) achieved blood glucose level control with the cutoff point of HbA_1c_ level <8% (Multimedia Appendix 1). Figure 1 shows the comparison of the cascades of care by setting with the overall cascade of care.

### Participant Characteristics

As can be seen in Table 3, the majority of our study participants with T2D were those aged ≥50 years (476/560, 85%), female (418/560, 74.6%), and with low educational level (primary school level or lower: 490/560, 87.5%). Of the 560 participants living with T2D, 251 (44.8%) were undiagnosed. In this bivariate analysis, we observed that age (*P*<.001) and sex (*P*=.03) were associated with the undiagnosed status of participants living with T2D.

**Table 3:** Characteristics of the diagnosed and undiagnosed type 2 diabetes cases in 2020 in Cambodia.

Characteristics		Overall (N=560), n (%)	Diagnosed cases (n=309), n (%)	Undiagnosed cases (n=251), n (%)	*P* value	
**Age (years)**						*<.001^a^*
	40-49	84 (15)	29 (9.4)	55 (21.9)		
	50-59	219 (39.1)	125 (40.4)	94 (37.5)		
	≥60	257 (45.9)	155 (50.2)	102 (40.6)		
**Sex**						*.03*
	Male	142 (25.4)	67 (21.7)	75 (29.9)		
	Female	418 (74.6)	242 (78.3)	176 (70.1)		
**Marital status**						.53
	Married or living with spouse	373 (66.6)	204 (66)	169 (67.3)		
	Widowed or not living with spouse	179 (32)	102 (33)	77 (30.7)		
	Never married and never lived together	8 (1.4)	3 (1)	5 (2)		
**Educational level**						.53
	No formal education or less than primary education	164 (29.3)	95 (30.8)	69 (27.5)		
	Primary education	326 (58.2)	179 (57.9)	147 (58.6)		
	Secondary education or higher	70 (12.5)	35 (11.3)	35 (13.9)		
**Household wealth quintile**						.45
	1 (poorest)	100 (17.9)	48 (15.5)	52 (20.8)		
	2	102 (18.2)	54 (17.5)	48 (19.1)		
	3	114 (20.3)	67 (21.7)	47 (18.7)		
	4	113 (20.2)	67 (21.7)	46 (18.3)		
	5 (richest)	131 (23.4)	73 (23.6)	58 (23.1)		
**Care initiative**						.22
	Coexistence of care	102 (18.2)	51 (16.5)	51 (20.3)		
	Community-based care	112 (20)	58 (18.8)	54 (21.5)		
	Health center–based care	117 (20.9)	60 (19.4)	57 (22.7)		
	Health center–based care with context	103 (18.4)	62 (20.1)	41 (16.3)		
	Hospital-based care	126 (22.5)	78 (25.2)	48 (19.2)		

^a^Italicization indicates values that met the significance threshold (*P*<.05).

In the multiple logistic regression analysis (Table 4), being aged 40 to 49 years was associated with higher odds of not receiving the T2D diagnosis (adjusted odds ratio [AOR] 3.2, 95% CI 1.9-5.5; *P*<.001) compared with those aged ≥60 years. Male participants with T2D displayed higher odds of not being diagnosed (AOR 1.7, 95% CI 1.1-2.5; *P*<.001) than female participants living with T2D.

We also observed that being in the poorest category of the wealth quintile was associated with having higher odds of not being diagnosed with T2D (AOR 2.3, 95% CI 1.3-4.2; *P*=.005) than those in the richest category. Finally, the care initiative setting was also associated with the undiagnosed status of participants with T2D. Compared with those in the hospital-based care setting*,* higher odds of not being diagnosed were observed in the coexistence of care setting (AOR 1.9, 95% CI 1.1-3.3; *P*=.03), community-based care setting (AOR 1.9, 95% CI 1.1-3.3; *P*=.02), and health center–based care setting (AOR 2.1, 95% CI 1.2-3.6; *P*=.01).

It was observed that among the 309 participants diagnosed with T2D, 177 (57.3%) were diagnosed by a private provider, 121 (39.2%) by a public provider, and 11 (3.6%) by others. Table 5 compares public providers with private providers in each setting in terms of the proportion of participants with diagnosed T2D status and that of those with T2D control status. No statistical significance was observed.

**Table 4:** Factors associated with the undiagnosed status of participants with type 2 diabetes in 2020 in Cambodia.

Characteristics		Adjusted odds ratio (95% CI)	*P* value
**Age (years)**			
	40-49	3.2 (1.9-5.5)	*<.001^a^*
	50-59	1.2 (0.8-1.8)	.34
	≥60	Reference	N/A^b^
**Sex**			
	Male	1.7 (1.1-2.5)	*<.001*
	Female	Reference	N/A
**Household wealth quintile**			
	1 (poorest)	2.3 (1.3-4.2)	*.005*
	2	1.5 (0.9-2.6)	.14
	3	1.1 (0.6-1.8)	.84
	4	1.0 (0.6-1.8)	.92
	5 (richest)	Reference	N/A
**Care initiative setting**			
	Coexistence of care	1.9 (1.1-3.3)	*.03*
	Community-based care	1.9 (1.1-3.3)	*.02*
	Health center–based care	2.1 (1.2-3.6)	*.01*
	Health center–based care with context	1.2 (0.7-2.0)	.60
	Hospital-based care	Reference	N/A

^a^Italicization indicates values that met the significance threshold (*P*<.05).

^b^N/A: not applicable.

**Table 5:** Distributions of participants with diagnosed type 2 diabetes (T2D) status and those with T2D control status categorized by health care provider in each setting.

Setting				Public provider, n (%)		Private provider, n (%)			*P* value	
**Participants with diagnosed T2D status (n=298^a^)**										.10
	Coexistence of care		26 (8.7)			22 (7.4)				
	Community-based care		16 (5.4)			41 (13.8)				
	Health center–based care		22 (7.4)			37 (12.4)				
	Health center–based care with context		27 (9.1)			35 (11.7)				
	Hospital-based care		30 (10.1)			42 (14)				
**Participants with** **T2D control status**										
	**Coexistence of care (n=38)**								.57	
		HbA_1c_^b^ level <8%	3 (7.9)		3 (7.9)					
		HbA_1c_ level ≥8%	12 (31.6)		20 (52.6)					
	**Community-based care (n=53)**								.88	
		HbA_1c_ level <8%	3 (5.7)		3 (5.7)					
		HbA_1c_ level ≥8%	25 (47.1)		22 (41.5)					
	**Health center–based care (n=55)**								.20	
		HbA_1c_ level <8%	1 (1.8)		10 (18.2)					
		HbA_1c_ level ≥8%	12 (21.8)		32 (58.2)					
	**Health center–based care with context (n=57)**								.23	
		HbA_1c_ level <8%	8 (14)		7 (12.3)					
		HbA_1c_ level ≥8%	15 (26.3)		27 (47.4)					
	**Hospital-based care (n=67)**								.19	
		HbA_1c_ level <8%	11 (16.4)		11 (16.4)					
		HbA_1c_ level ≥8%	15 (22.4)		30 (44.8)					

^a^The category of *other provider* was removed from the analysis owing to its small proportion, which made the statistical test unreliable.

^b^HbA_1c_: glycated hemoglobin.

## Discussion

### Principal Findings

This study used primary data from a cross-sectional survey to generate the cascade of care for the T2D care continuum in 5 purposively selected ODs in Cambodia. Overall, the cascade displayed substantial drops at the testing stage (207/560, 37%, loss from the *prevalence* bar) and at the control stage (213/560, 38%, loss from the *in treatment* bar), indicating that all selected settings, regardless of the care initiatives present, have limited capacity to detect people with T2D and control the condition (blood glucose level control) in those with T2D despite being in receipt of treatment. The findings were consistent with the T2D cascade analyses in other LMICs that displayed significant losses at the testing stage (also 37%) [18]. However, the drop between the treatment stage and the control stage observed in this study was much larger than that in the studies in other LMICs—only 15% in other LMICs compared with 38% (213/560) in this study [18]. With the cutoff point of HbA_1c_ level <8%, the proportion of those with T2D under control was 23% in other LMICs [18]; in this study, 10.7% (60/560) were considered as having achieved blood glucose level control. This is an exceptionally low rate, indicating that T2D in Cambodia is not being treated properly and adequately.

We disaggregated the cascades of care by study setting to observe the influence of the care initiatives. Unexpectedly, the coexistence of care setting displayed the worst cascade across all bars, whereas the hospital-based care setting had the best cascade among the 5 settings. This discovery was unexpectedly contradictory to the ICCC theoretical framework [3], calling into question the assumption underlying the ICCC framework that the combined care initiatives of health care organization and community represent an ideal context for integrated care for T2D and thereby would reduce leakages in the cascade. This suggests that the presence of health care infrastructure is not directly translated into improved care performance [29]. Implementation fidelity that focuses on the process of care implementation has to be taken into account [30]. Working mechanisms such as integrated care management across care levels and actors, the use of shared disease registries, and coordinated resources for self-management support and community education have to be in place for the coexistence of care to represent the ideal ICCC framework [3,16]. An investigation of the actual implementation of the care initiatives in these ODs was conducted in another study (Te V et al, unpublished data, July 2022). The investigation found that the 3 care initiatives were not implemented in an integrated way as intended in the written guideline [11] but in isolation, with limited interaction among them. The working mechanisms that facilitate integrated care for T2D in terms of shared necessary information and coordinated resources [16] were not observed. There was no proper system for following up patients for the continuity of care. The referral system among the communities, health centers, and referral hospitals was dysfunctional. The peer educator network in the OD with coexistence of care was not functioning optimally. The network had been handed over to the OD health authorities for governance, and technical or financial support from MoPoTsyo disappeared, rendering the network dysfunctional.

It should be noted that the care initiatives were not solely responsible for the provision of care for T2D in the selected ODs. On the basis of the same survey data, we found that, in general, health care use occurred dominantly in the private sector (78% among those seeking care in the 3 months preceding the survey), and referral hospitals were the common public health care facilities used by those with T2D and hypertension [31]. Therefore, our findings may not be fully attributed to the care initiatives. In the community-based care setting of OD Kong Pisey, only 12% (7/58) of the study participants were people living with T2D who were connected to the peer educator network, and only 4% (2/51) were connected to the peer educator network in the coexistence of care setting of OD Daunkeo. This may potentially undermine the effectiveness of community-based care. In a study based on MoPoTyso’s routine data, 43% of the people in the network achieved the median HbA_1c_ level of 7.1% [32]. In the hospital-based care setting of OD Samrong, only 5% (4/78) of the participants were seeking T2D care or treatment at the NCD clinic of the referral hospital in the 3 months preceding the survey, whereas at WHO PEN health centers in the health center–based care setting of OD Pearaing with high WHO PEN coverage, 3% (2/60) of the participants were identified seeking care for T2D.

Further statistical analysis, although not statistically significant, found that in all settings, except for the coexistence of care, private providers—who could not be fully incorporated into our study design owing to a lack of trustworthy information system in this sector—played a dominant role in diagnosing people with T2D. This suggests that the coexistence of care would increase the role of public health providers in the care continuum. In another study based on the same survey data, we found that the proportion of people with T2D seeking care at public health care facilities was higher than that of those with only hypertension or no condition [31]. This increased use of public health care facilities was also associated with a reduction in health care expenditure among patients, especially those in the poorest category of the wealth quintile who benefit from Health Equity Fund membership [31]. In a health system–level study, financial constraints have been found to be one of the main barriers to the T2D care continuum [6].

In this study, we found that 11.04% (560/5072) of the participants aged ≥40 years were identified as having T2D—of whom almost half (251/560, 44.8%) had not been diagnosed. This is a high prevalence rate because the overall prevalence rate of undiagnosed T2D in other LMICs has been reported to be 4.8% [18]. Predictors of being undiagnosed were being aged 40 to 49 years, being male, or falling in the poorest category of the wealth quintile. This suggests that more testing efforts are needed from the health system to reach people at risk for T2D, especially those from the aforementioned groups. A systematic review found that targeted screening was more cost-effective than universal screening [33]. A more convenient implementation arrangement for immediate diagnosis after testing should be put in place so that avoidable loss between these stages can be further minimized. In the national standard operating procedure [11], the WHO PEN health center staff are only allowed to perform the screening, whereas the diagnosis needs to be confirmed by the physician at the NCD clinic of the referral hospital. If the people who have been screened cannot have access to the diagnosis procedure at the referral hospital for some reason, the chances of not receiving prompt care or treatment increase. This requires strong coordination between the health centers and the NCD clinics, which has to be robust and supportive.

### Limitations

First, despite using the primary data collected intentionally for the construction of the cascades of care, the sample size was not large enough to yield a sufficiently large number of patients with T2D who had achieved T2D control to enable us to assess the determinants of this particular bar. Second, the care initiatives, either individually or in coexistence, were not exclusively responsible for the provision of T2D care in each OD, thereby resulting in a weak connection between the presence of care initiatives and the cascade of care results of each study setting. We used the OD as a proxy variable to measure the effect of the care initiative, which in fact could mask a number of potential confounding contextual factors such as the dominant use of private services. In addition, in the Cambodian health system, the population is not confined to a particular public health facility in the catchment area. People can shop around freely, which means that patients may use services outside the catchment area of the facility. Third, the cross-sectional design did not allow us to determine the causal pathways leading to diabetes care outcomes, and the use of self-reported data in related sections could have produced biased results. A longitudinal study design with the collection of routine cohort data would enable us to address the limitations and evaluate the effectiveness of the different T2D care initiatives over time. This can be supported by digital health interventions. Systematic reviews have demonstrated the effectiveness of telemedicine via smartphone functions to provide self-care education, facilitate self-monitoring, produce the required treatment reminders, and collect feedback for health care professionals, which facilitates informed treatment recommendations [34,35]. In Cambodia, a study assessing the potential use of a wearable health monitor in the prevention and control of NCDs revealed that this health technology had the potential to support activities related to health promotion, patient follow-up and monitoring, and surveys of NCD risk factors, with positive user experiences and high levels of acceptance [36,37]. A digital health intervention that was tried among the MoPoTsyo networks produced valuable knowledge on pathways to address barriers to successful adoption in the Cambodian context [38].

### Conclusions

This study provided an updated estimate of T2D prevalence among people aged ≥40 years (approximately 1 in 10 people) in Cambodia. The findings revealed that the unmet need for T2D care was large, particularly in the testing and control stages, indicating the need to substantially improve early detection and management of T2D in Cambodia. With almost half of the study participants with T2D undiagnosed (251/560, 44.8%) and thus unaware of their condition, early detection of people with T2D is an important first step that the health system needs to achieve to improve the T2D care continuum. We recommend rapid scale-up of T2D care components at public health facilities to increase the chances of the population with T2D of being tested, diagnosed, retained in care, and treated, as well as of achieving blood glucose control. At the same time, raising awareness and encouraging testing among the population at risk through a broad public health campaign should be one of the priorities. With advanced technology, a social media campaign has the potential to reach large parts of the population at low cost. Public health care use can reduce financial constraints among the population, particularly among those in the poorest category of the wealth quintile. We also recommend that within the context of resource constraints, specific groups considered susceptible (being male, being aged 40-49 years, or falling in the poorest category of the wealth quintile) should be especially targeted for testing through active community outreach activities because these groups are more likely to be unaware of their T2D condition. Adding care during off-hours for chronic conditions, including T2D, at public health facilities could increase access to care for male patients who are employed or busy during working hours. Future research should focus on evaluating the effectiveness of the different T2D care initiatives longitudinally with more diverse population groups from various settings. Given that digital health interventions have the potential to improve the prevention and control of NCDs while, at the same time, collecting longitudinal routine data vital for integrated care, feasibility and effectiveness studies of digital health interventions, such as telemedicine and mobile health, should be prioritized as a promising means to enable improvements along the T2D care continuum in Cambodia.

## Appendix

Multimedia Appendix 1 Cascade of care for type 2 diabetes (T2D) in 2020 in Cambodia. HbA_1c_: glycated hemoglobin.

## Abbreviations

AOR: adjusted odds ratio
FBG: fasting blood glucose
HbA_1c_: glycated hemoglobin
ICCC: innovative care for chronic conditions
LMICs: low- and middle-income countries
NCD: noncommunicable disease
NECHR: National Ethics Committee for Health Research in Cambodia
OD: operational district
SCUBY: Scale-up Integrated Care for Diabetes and Hypertension
T2D: type 2 diabetes
WHO PEN: World Health Organization package of essential noncommunicable disease interventions
WHO: World Health Organization

## Data Availability

The data sets generated and analyzed during this study are available from the corresponding author on reasonable request.
