# Supplementary figures and images for: Evaluation of Diabetes Care Performance in Cambodia Through the Cascade-of-Care Framework: Cross-Sectional Study
Source: JMIR Public Health Surveill. 2023 Jun 22;9:e41902. doi: 10.2196/41902 (PMC10337437; doi:10.2196/41902)

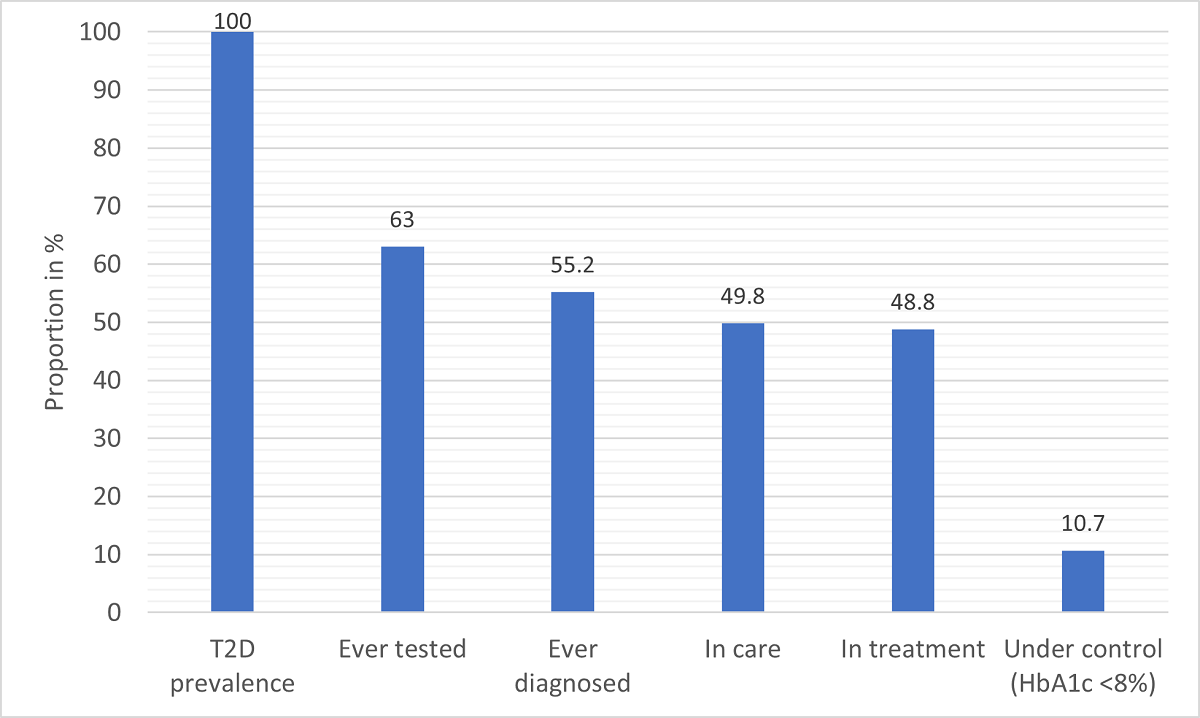

Supplement: Multimedia Appendix 1 [file publichealth_v9i1e41902_app1.png]
